# Post-COVID Multisystem Inflammatory Syndrome-Adult Leading to Cardiomyopathy and Autoimmune Thyroiditis: A Case Report

**DOI:** 10.7759/cureus.33754

**Published:** 2023-01-13

**Authors:** Varsha R Bhatt, Pranav G Jawade, Aditi M Patel, Amit Palange, Saimounika Adapa

**Affiliations:** 1 General Medicine, Dr. D. Y. Patil Medical College, Hospital & Research Centre, Pune, IND

**Keywords:** sars-cov-2, mis-a, auto-immune thyroiditis, corona virus cardiomyopathy, covid-19

## Abstract

The Coronavirus disease pandemic is an evolving disease with myriad presentations and sequelae. Multisystem inflammatory syndrome in adults (MIS-A) can affect various organ systems, including cardiovascular, gastrointestinal, and neurologic systems, with fever and abnormally increased inflammatory markers without significant respiratory affection. This is a well-known complication in children (MIS-C). Validated clinical criteria are used to diagnose this condition. Long-term sequelae of MIS-A are unclear and underreported. Here, we describe a case of Post-COVID-19 MIS-A who presented with cardiac dysfunction, hepatitis, and acute kidney injury and recovered well with steroids. He was left with persistent cardiomyopathy and thyroiditis with hypothyroidism which to date has not fully recovered. This case emphasizes that the sequelae of COVID-19 and its pathophysiology are not fully understood, and more research is needed to predict and prevent the same.

## Introduction

The ongoing pandemic of COVID-19 has presented immense challenges for the medical fraternity and scientists. Several manifestations during and after COVID-19 infection make it challenging to manage. Several extra-pulmonary manifestations are also being reported with COVID-19. Recently, a unique phenomenon in the form of Multisystem Inflammatory Syndrome (MIS) has been reported worldwide, affecting children and adults. Multiple organs and systems have been involved due to a dysregulated immune response, with constitutional symptoms, elevated inflammatory markers, and the absence of overt pulmonary symptoms, constituting a multisystem inflammatory syndrome [1]. While many such cases have been reported in the literature in the last two years, relatively fewer data is available on residual post-Multisystem Inflammatory Syndrome-Adult (MIS-A) cardiomyopathy and thyroid involvement. According to the literature, 3.56 per 1000 COVID-19 patients developed non-ischemic cardiomyopathy, but MISA-A was not reported [2].To our knowledge, the literature has not described persistent post-MIS-A cardiomyopathy. One case report of post-COVID Hashimoto's thyroiditis has been described [3]. We believe that literature related to post-COVID complications is continuously evolving. Hence, we report a case of a male in his thirties who presented with MIS-A. After the acute phase, he developed residual post-MIS-A cardiomyopathy and thyroiditis, leading to hypothyroidism. This case prompts physicians to look for rarer long-term sequelae of post-COVID MIS-A.

## Case presentation

A male in his thirties with no known co-morbidities and no addictions came to a tertiary care center in Western India, complaining of fever and nocturnal dry cough for two days with breathlessness for three days (grade 2 Modified Medical Research Council Dyspnea scale). He also complained of right hypochondriac pain for the last four days, which was localized, non-radiating, and associated with nausea and dullness. He had a decreased urine output for two days. The patient had a history of fever, cough, and cold two weeks before this presentation and did not consult a doctor. He was unvaccinated for COVID-19. He had undergone routine health checks every six months, giving him a clean bill of health, including normal thyroid functions.

On examination, he was moderately built and nourished. The patient's temperature was 101.6 Fahrenheit on admission. He was hemodynamically stable, with a respiratory rate of 28 breaths per minute. Oxygen saturation (Sp02)was 90% in room air and required 2 liters of oxygen via nasal prongs. He had mild pallor, mild icterus, and bilateral pitting pedal edema. Cyanosis, enlarged lymph nodes, and clubbing was not present. On cardio-vascular examination, his first heart sound was soft, and a third heart sound was heard. A grade 3/6 pansystolic murmur was heard in the mitral area, radiating to the axilla. Extensive bilateral crepitations in the infra-scapular, infra-axillary, and inter-scapular regions were heard on respiratory system examination. There was mild tenderness in the right hypochondriac region, and hepatosplenomegaly was appreciated. Upon neurological examination, the patient was conscious and oriented. No motor or sensory deficits were seen. The patient was admitted to the intensive care unit and further underwent a series of investigations.

**Figure 1:**
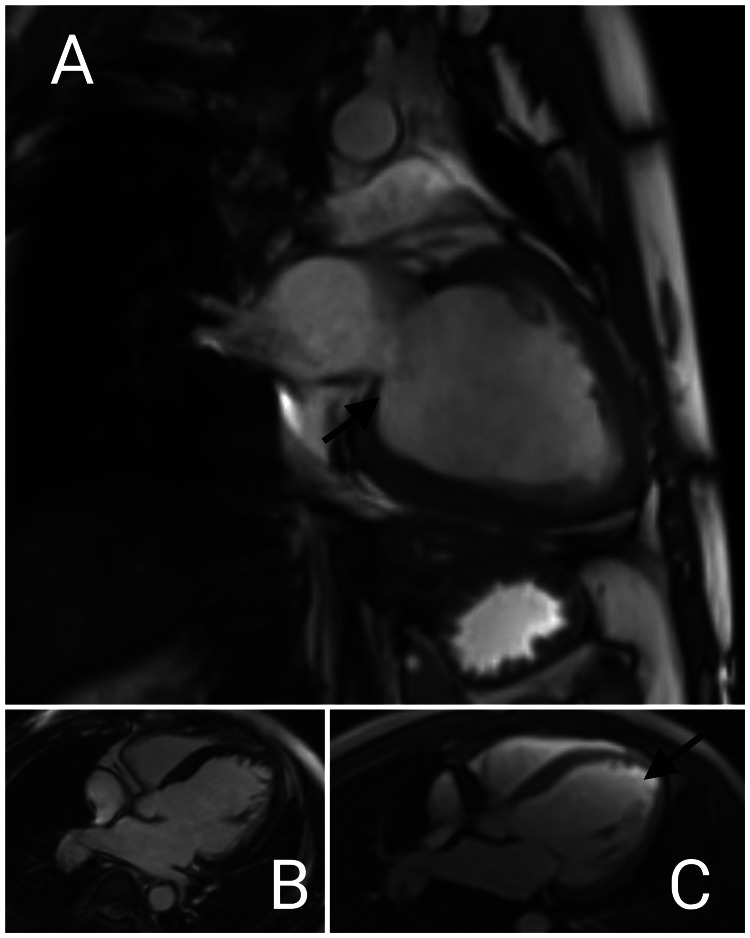
A: Bright Blood 2 chamber long axis image showing dilated left ventricle with mild Mitral regurgitation. B: 4 chambers first past contrast image showing dilated left atrium, ventricle with no thrombus. C: 4 chamber Phase-Sensitive Inversion-Recovery (PSIR) contrast image showing no myocardial enhancement.

**Figure 2:**
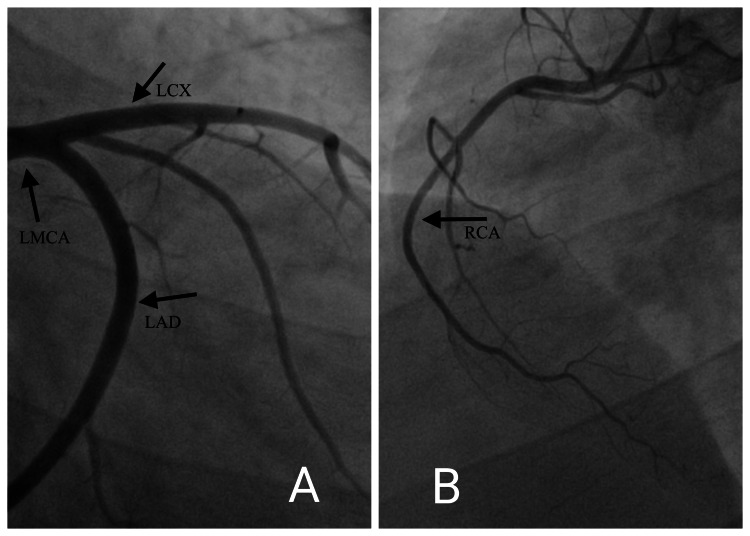
A: Coronary Angiogram showing normal Left Main Coronary Artery (LMCA), Left Anterior Descending Artery (LAD), and Left Circumflex Artery (LCX). B: Coronary Angiogram showing normal Right Coronary Artery (RCA).

The patient's electrocardiogram was normal. Chest X-ray was suggestive of cardiomegaly, bilateral lower zone haziness, bilateral cardiophrenic angle obliteration, mildly bulky bilateral hilum, and prominent vascular markings suggestive pulmonary edema. HRCT thorax with interlobular septal thickening in the central portion of bilateral lung fields with peripheral spacing, Kerley lines, Bilateral pleural effusion, and cardiomegaly, which was suggestive of pulmonary edema. Ultrasound of the abdomen and pelvis was suggestive of hepatomegaly and bilateral pleural effusion with minimal ascites. 2-dimensional echocardiography on the day of admission was suggestive of an ejection fraction of 30% with global Left Ventricular hypokinesia (anterior > inferior), moderate Mitral Regurgitation, moderate Tricuspid Regurgitation, moderate Pulmonary Artery Hypertension (Right Ventricular Systolic Pressure- 48 mm hg). The patient underwent a USG Neck that was suggestive of a heterogenous and normal-sized thyroid. The patient's Cardiac Magnetic Resonance Image (Figure 1) was suggestive of moderately dilated cardiomyopathy with a dilated left ventricle and mild mitral regurgitation. His coronary angiogram (Figure 2) was suggestive of normal left main coronary artery (LMCA), left anterior descending artery (LAD), and left circumflex artery (LCX) along with right coronary artery (RCA), thereby ruling out any ischemic event.

The patient's SARS-CoV-2 Reverse Transcriptase -Polymerase Chain Reaction (RT-PCR) was sent, which was negative. However, SARS CoV-2 total antibodies report was positive [81,360 reference interval, 1000 index (S/C)] with SARS CoV-2 IgM positive. On admission patient's complete Blood count was normal. His renal function test was deranged with a creatinine of 2.5 mg/dl and urea of 74 mg/dl. The liver function tests were deranged with alanine transaminase of [944 U/L; reference interval 7 to 55 U/Lt] and aspartate transaminase of [1138 U/L; reference interval 8-48 U/Lt]. The patient's Inflammatory markers were as follows C-Reactive Protein- [16.2 mg/L reference interval low cardiovascular risk <2.0 mg/L], Ferritin- [794.12 ng/ml; male reference interval 21.81-274.66], d-dimer- 1612 ng/ml, which increased up to [>10,000 ng/ml; reference interval 0 to 500 ng/ml] on the fifth day of admission in subsequent days and serum Lactate Dehydrogenase [1674 U/L; reference interval 85 to 227 U/Lt]. The patient's Troponin I level was normal, and Creatinine Kinase MB (CKMB) was [78 IU/mL; reference interval < 24 U/Lt] interleukin-6 levels were done on day three of admission and were high at [12.94 pg/mL; reference interval < 7 pg/mL] The patient thyroid profile showed an elevated Thyroid Stimulating Hormone (TSH) of [37.25 µIU/mL; reference interval 0.35 to 4.94 µIU/mL, normal T3 and T4, but positive Anti-Thyroid Peroxidase antibodies [191.69 IU/mL; reference interval <5.61 IU/mL] (table 1). The patient was non-diabetic and was negative for dengue, typhoid, Leptospira, and Malaria serologies; in such a scenario, diagnosis of post-COVID-19, Multisystem inflammatory syndrome-Adult was considered as he fulfilled the validated clinical criteria. The patient's blood and urine cultures showed no growth of any micro-organisms.

**Table 1:** Laboratory results of the patient Anti-TPO antibodies: anti-thyroid peroxidase antibodies  TSH: Thyroid Stimulating Hormone LDH: Lactate Dehydrogenase

TEST	RESULT	REFERENCE RANGE
SARS CoV-2 RTPCR	Negative	
SARS CoV-2 Total Antibodies	IgM Positive	
Serum Creatinine	2.5 mg/dl	0.6 to 1.2 mg/dl
Serum Urea	74 mg/dl	17 to 49 mg/dl
Alanine Transaminase	944 U/L	7 to 45 U/Lt
Aspartate Transaminase	1138 U/L	8 to 43 U/Lt
C-reactive Protein	16.2 mg/L	<2 mg/L- low cardiovascular risk
Serum Ferritin	794.12 ng/mL	21.81 to 274.66 ng/mL
Serum LDH	1674 U/L	85 to 227 U/L
D-dimer	1612 ng/ml	0 to 500 ng/ml
TROPONIN-I	<10 pg/ml	<10 pg/ml
Creatine Kinase-MB	78 IU/mL	up to 24 IU/mL
Interleukin-6	12.94 pg/mL	<7 pg/mL
Serum TSH	37.25 µIU/mL	0.35 to 4.84 µIU/mL
Anti-TPO antibodies	191.69 IU/mL	<5.61 IU/mL
Blood culture and sensitivity	No Growth	
Urine culture and sensitivity	No Growth	

The patient received treatment with Intravenous Furosemide, supplemental oxygen, and anti-platelet drugs like aspirin (75 mg once daily) and clopidogrel (75 mg once daily). In a dose of one gram, Intravenous methylprednisolone was given for three days, followed by 80 mg Intravenous, and then slowly tapered over 14 days. Digoxin (0.25mg once a day for five days a week), sacubitril-valsartan (50mg twice daily) and rivaroxaban (10mg once a day), and L-thyroxine (100 mcg daily) were added. The patient was discharged on aspirin, L-thyroxine, torsemide, carvedilol, sacubitril-valsartan, and rivaroxaban for a month. The patient's informed consent was taken. On follow-up, the patient's ejection fraction improved to 40% but did not improve further for the following year. He was symptomatically better and became euthyroid after six months of treatment. He is currently on aspirin, torsemide, sacubitril-valsartan, and L-Thyroxine.

## Discussion

Multisystem inflammatory syndrome in adults is a known post-COVID-19 complication. It was reported widely in the pediatric population after the second wave in several parts of the world as Multisystem Inflammatory Syndrome Children (MIS-C). MIS-A is undoubtedly a late manifestation of abnormal immune response to SARS CoV-2 infection in adults manifesting into a multi-systemic inflammatory immune-mediated damage that involves cardiovascular, hepatic, gastrointestinal, renal, vascular, endocrine, etc., systems [4-6].

The Centers for Disease Control and Prevention (CDC) has stated the following five criteria to establish the diagnosis of MIS-A: (1) concurrent or previous (within the past 12 weeks) COVID-19 diagnosed by either PCR or antigen/antibody testing, (2) severe sickness necessitating hospitalization in those aged 21 years or more, (3) marked involvement or dysfunction of single or multiple extra-pulmonary organs (acute kidney injury, acute liver injury, neurological involvement, cardiac insult, shock, hypotension, and so on), (4) absence of severe respiratory affection (respiratory signs and symptoms), and (5) exhibiting severe inflammation as per laboratory findings: elevated C Reactive Protein, d-dimer, serum ferritin, erythrocyte sedimentation rate (ESR), fibrinogen, interleukin-6 (IL-6) [7]. Our patient fulfilled the required criteria for establishing the diagnosis of MIS-A. The pathophysiology of MIS-A is unknown to a great extent. A case series by Magro et al. in the year 2020 discusses the pathology of MIS-A with features of endotheliitis and deposition of complement in the vessels [8].

According to Behzadi et al., in an article published in 2022, out of 36 reported MIS-A cases documented, 17 cases had COVID-19 infection confirmed via RT-PCR, antibody tests, and clinically [9]. In the case series published on October 2020 by Morris et al., it was found that a significant number of cases (8/27) of MIS-A had negative Polymerase Chain Reaction (PCR) and positive SARS CoV-2 antibody test results, thereby suggesting that MIS-A might represent a post-infectious process [7]. Extra-Pulmonary manifestations of COVID-19 infection include endothelial damage, thrombo-inflammation, dysregulated immune response, and dysregulation of the Renin Angiotensin Aldosterone System [6]. Several articles on MIS-C and MIS-A found that several cases came with an RTPCR negative report. COVID antibody testing must be done concurrently to confirm the diagnosis of previous COVID-19 infection [7,10,11].

In our case, post-MIS-A cardiomyopathy, which lasted almost a year after his illness, was another significant aspect. In Wuhan, out of 44,672 cases of COVID-19, cardiovascular impairment, like heart failure, was seen in 5% of the cases during and after the illness [12-13]. In various cases of MIS-A, cardiovascular pathology and symptoms were remarkable, including cardiogenic shock and heart failure with reduced ejection fraction [14-16]. Some cases have been reported as post-COVID-19 myocarditis but no preceding MIS-A [17]. In his article published in the year 2022, Abbasi et al. found that 12 months past the pandemic, in comparison with the control group, for every 1000 people, COVID-19 was associated with extra 23.48 incidents of major adverse cardiac events, including myocardial infarction, stroke, etc., 19.86 incidents of dysrhythmias predominantly atrial fibrillation and 11.61 incidents of heart failure and 3.56 incidents of non-ischemic cardiomyopathy; 7.28 incidents of ischemic heart disease. Also, SARS CoV-2 infection has led to de-novo cardiovascular disorders [2]. A study on MIS-A by Hékimian G et al. in the year 2021 found that most subjects had positive antibody serology tests on admission with negative RT-PCR reports, suggesting the hyperinflammatory response and immune-mediated systemic and cardiac damage in MIS-A [18]. However, persistent cardiomyopathy after MIS-A has not been reported in the literature. According to Feghali et al. in their report published in 2021, SARS CoV-2 infection is rarely associated with autoimmune diseases like Hashimoto's Thyroiditis and Guillàn Barre Syndrome, and very little is known about its pathogenesis. Histology findings suggest the indirect, immune-mediated mechanism associated with destructive follicular thyroiditis and cellular apoptosis [3,19]. In our case, however, both cardiomyopathy and thyroiditis leading to hypothyroidism developed after MIS-A and not directly after acute COVID-19 infection. 

Treatment options for MIS-A include Intravenous Immunoglobulins, Intravenous corticosteroids, Tocilizumab, Extracorporeal Membrane Oxygenation and vasopressors, and various others. However, according to McArdle et al. in their article published in 2021, there is no evidence of higher recovery rates with Intravenous immunoglobulin, Immunomodulators, or a combination of the two [20].

## Conclusions

COVID -19 is an ever-evolving disease. MIS-A is a complication of COVID-19 due to dysregulated immune response, which leads to multiorgan affection. Significantly less is known of long-term sequelae of MIS-A in COVID-19. This case emphasizes persistent cardiomyopathy and autoimmune thyroiditis, which developed in a previously healthy male post-MIS-A. More research is needed to understand the immunological phenomena of COVID-19.
